# GPS and GPRS Based Telemonitoring System for Emergency Patient Transportation

**DOI:** 10.1155/2013/363508

**Published:** 2012-12-10

**Authors:** K. Satyanarayana, A. D. Sarma, J. Sravan, M. Malini, G. Venkateswarlu

**Affiliations:** ^1^Department of Biomedical Engineering, Osmania University, Hyderabad 500 007, India; ^2^Research and Training Unit for Navigational Electronics, Osmania University, Hyderabad 500 007, India; ^3^Department of ECE, Vasavi College of Engineering, Hyderabad 500 031, India

## Abstract

Telemonitoring during the golden hour of patient transportation helps to improve medical care. Presently there are different physiological data acquisition and transmission systems using cellular network and radio communication links. Location monitoring systems and video transmission systems are also commercially available. The emergency patient transportation systems uniquely require transmission of data pertaining to the patient, vehicle, time of the call, physiological signals (like ECG, blood pressure, a body temperature, and blood oxygen saturation), location information, a snap shot of the patient, and voice. These requirements are presently met by using separate communication systems for voice, physiological data, and location that result in a lot of inconvenience to the technicians, maintenance related issues, in addition to being expensive. This paper presents design, development, and implementation of such a telemonitoring system for emergency patient transportation employing ARM 9 processor module. This system is found to be very useful for the emergency patient transportation being undertaken by organizations like the Emergency Management Research Institute (EMRI).

## 1. Introduction

**Figure 1:**
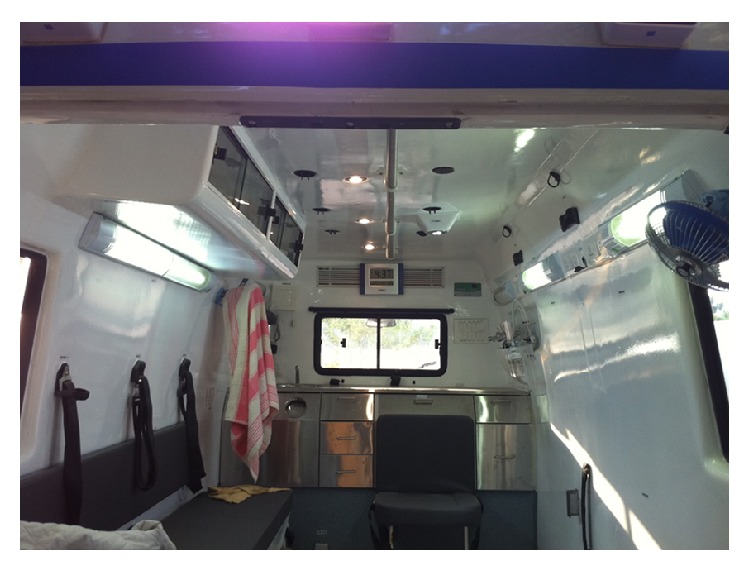
Interior view of a typical ambulance.

Immediate medical attention to critically ill patients and accident victims followed by transportation to a well-equipped medical facility within the golden hour saves many lives. Numbers of road accidents in India are the highest across the world. According to the National Transportation Planning and Research Center (NTPRC) the number of road accidents for 1000 vehicles in India is about 35 while the figure is between 4 to 10 in developed countries. About 1,05,000 accidents take place every year [1]. There are several governmental and nongovernmental agencies like the Emergency Management Research Institute (EMRI), located across the country, which have been dedicated to the cause of transporting critically ill patients and accident victims. About 2,87,000 lives have been saved by EMRI in the past six years. The ambulances are specially designed to carry emergency drugs and instruments. Inner area of ambulance is fabricated in such a way that it houses emergency medicines, sterilizer, stretcher, and so forth. A typical inside layout of an ambulance employed for emergency patient transportation is shown in Figure 1. The paramedics that accompany the ambulances are specially trained to be emergency technicians. There exists a need to augment the skill set of such paramedics with the expert doctor's advice from the central monitoring station (CMS). CMS helps in identifying the nearest and appropriate hospital and coordinating with the medical personnel of that hospital.

Hence there is a need for communication between the staff of the ambulance and the central monitoring station. The doctor needs to understand the physical and physiological condition of the patient so that the right decision regarding administration of drugs and transport destination can be appropriately taken. Administrative requirements include the location of the vehicle and personnel attendance details. Deploying these instruments would mean that separate communication mechanism, namely, the usage of separate SIM cards and separate GSM/GPRS modems, would be required resulting in increased recurring expenditure. Hence a comprehensive, cost effective system that can acquire physiological data from the patient keyed-in data from a keyboard and location data from a GPS receiver as well as voice signals and send them using cellular network is needed.

**Figure 2:**
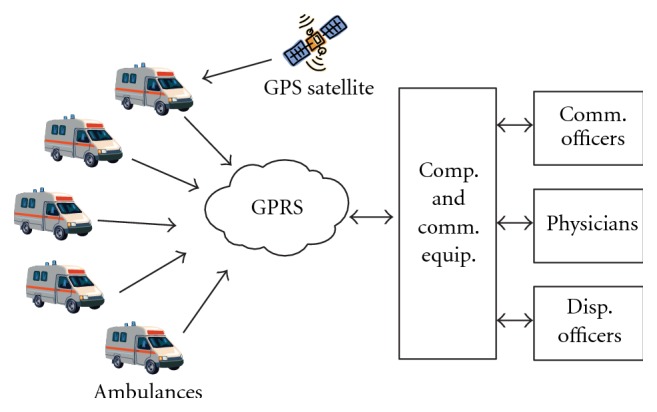
Architecture of the proposed system.

Several systems related to telemonitoring are reported in the literature [2]. Liszka et al. [3] and Plesnik et al. [4] have developed a real-time remote monitoring system for combining ECG data and GPS data before transmitting to a Central Monitoring Station (CMS). Similar systems are independently developed by researchers at the Glenn Research Center (NASA), University of Akron and Case Western University. Khan and Mishra [5] reported that position and velocity of vehicle can be estimated using GPS receiver fitted in the vehicle and sent to the central monitoring station using GSM. Zhang and Lu [6, 7] developed an ECG telemonitoring system with GPS and GPRS to continuously monitor ECG of the cardiac patient along with the position and posture. Fang and Lai [8] have developed a system to monitor the ECG of cardiac patient who is away from the hospital. A mobile ECG telemonitoring system along with an accelerometer, to sense sudden postural changes that reflect sudden cardiac failure, has been developed [9]. Philips Company has developed systems for remote monitoring of cardiac patients from their research centers worldwide in 2009. Exact and continuous blood pressure monitoring system along with location information and time synchronization facility to work with other measurement modules has also been proposed [10]. Recent advances in telemonitoring systems resulted in early diagnosis and management of chronic and degenerative conditions, significantly prevalent in elderly people. The number of recurring visits to the hospital can be reduced. Physicians are provided with better insight into the patient's health. Telemonitoring can also be applied on a long-term basis to elderly persons to detect gradual deterioration in their health status resulting in inability to live independently. Monitoring the activity of upper limbs can help in assessing the health status [11]. Several digital signal processing techniques have also been employed to denoise and study GPS and ionospheric studies [12, 13]. Companies including QRS diagnostic, VivoMetrics, Human Network Technology, AMD Telemedicine, Health Frontier, and CardioNet have been engaged in the development of telemonitoring systems. Though all these systems are intended towards remote monitoring of cardiac patients, the concept of a unified system is not satisfied by any of the existing systems. They do not cater to the specific requirement of acquiring the physiological data, keyed-in data, patient snapshot, and voice signals and transmitting them using cellular network. Hence, it is proposed to develop an indigenous system to meet these requirements (Figure 2). The system developed by us is a comprehensive, cost-effective system (approximately USD 4500), which can acquire the physiological data, GPS data, vehicle parameters, patient information, patient snap shot, and SOS messages and transmit them as a single data packet using a cellular network. 

This system has been built around ARM 9/11 microcontroller based module exclusively designed for this purpose and integrated with commercially available GPS and GSM modules and physiological signal acquisition systems like Medicaid and GSM phone with Blue Tooth (BT) facility. The necessary software for the embedded system has been developed using KEIL compiler for ARM family of microcontrollers. The front end application at the central monitoring system is developed using.net technologies. The system has been deployed in an ambulance for initial evaluation. This system is found to be very useful for emergency patient transportation being undertaken by organizations like EMRI.

## 2. Design Aspects of the System

The designed and developed ambulance system consists of GPS receiver, signal and image acquisition system, GSM/GPRS modem, and microcontroller unit. The central monitoring system has a bank of GPS/GPRS modems, central server and application software. Hardware design challenges include combining different commercial off-the-shelf (COTS) devices and specifically built subsystems, design, and implementation of interprocessor communication interfaces. Software design challenges include different sampling rates on separate channels to meet the Nyquist sampling rate of signals with varying bandwidths and reducing the data packet size. Implementation of interprocessor communication protocols is also complex. The typical specifications of the proposed system are as shown in Table 1.

## 3. Ambulance Electronics Unit

**Figure 3:**
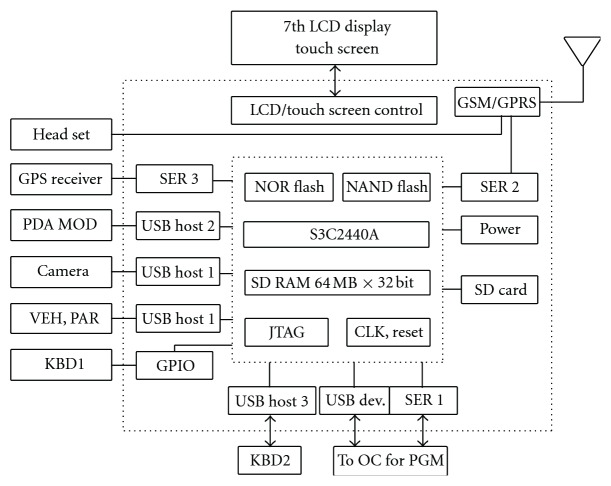
Architecture of ambulance electronics system.

The electronics system present in the ambulance consists of a powerful low cost 32 bit RISC based microprocessor based system. It acquires location, speed, and time data from GPS receiver and physiological signals from an exclusively developed and built medical data acquisition module. Image from a camera, administrative data from a keyboard, and vehicle parameters from a vehicle data module are also acquired (Figure 3). All these data are combined to form a data packet, in predefined format, and transmitted using GPRS module to a central monitoring station. The central monitoring station houses call center personnel as well as trained medical personnel and expert doctors, who analyze the signals and data coming from field ambulances. 

### 3.1. Microprocessor Module

The heart of the ambulance unit is built around ARM 9 Samsung processor S3C2440A operating at 400 MHz. The unit consists of two major building blocks, namely, processor module and base module. The processor module and base module are separately designed to provide flexibility and reduction in cost. Processor module consists of 6 layers whereas base module is a double layered one. The S3C2440A has ARM920T core. It adopts a new bus architecture known as Advanced Microcontroller Bus Architecture (AMBA). The integrated on-chip functions include SDRAM Controller, LCD Controller, 4-channel DMA Controllers, 3-channel UARTs, 2-channel SPI, IIC, IIS, Audio AC′97, 2-channel USB Host Controller, Camera Interface, and RTC Calendar function. Processor module includes nonvolatile NOR Flash of 2 MB (SST39VF1601), nonvolatile NAND Flash of 256 MB (K9F2G08U0B), and 32 bit SDRAM of 64 MB (2 X HY57V561626FTP-H), operating at a clock frequency of 100 MHz. NOR flash houses BIOS routines for interfaces and resources. The activities of microcontroller are indicated by three LEDs mounted on the board. NAND flash houses KERNEL, OS, and file system. SDRAM is used for temporarily storing the data for processing.

### 3.2. Base Board Module

**Figure 4:**
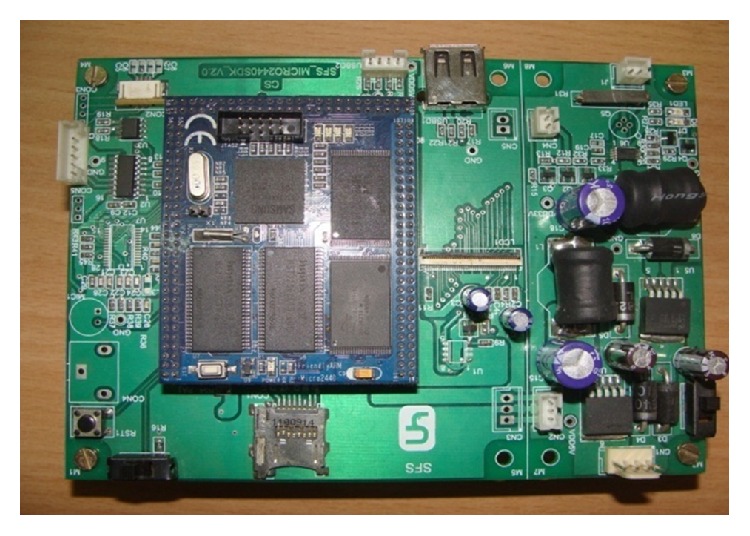
Photograph of the base board with core module.

Baseboard houses key board, programming interfaces, memory card, and audio devices. Alcor micromake USB hub IC (AU9254A) enhances the number of USB host ports to 4. USB host 1 connects camera and USB host 2 connects PDA system; USB host ports 3 and 4 are used for connecting external keyboard and vehicle parameter modules, respectively. USB device port helps in connecting to PC for downloading programs and OS images from the computer. There are three serial ports provided. Serial port 1 is connected to PC for programming purpose. Serial port 2 is connected to GSM/GPRS/3G modem for transmitting data, voice, and images. Serial port 3 is connected to GPS receiver to collect Position, velocity, and time (PVT) information at a speed of 4800/9600 bps. The data is updated every second. Transcend make Micro-SD card with necessary SD/MMC interface (clock, command, and 4 data lines) with a storage capacity of 4 GB is used for local storage of data and housing related file system. 10/100 MBPS LAN connectivity is provided with Ethernet IC DM9000. Audio CODEC Philips make UDA1341TS provides sound functionality of the system. The core module is connected to the base board using three 50 pin connectors. This board also connects LCD module through 40 pin flat cable. General purpose I/O pins are terminated in a connector for future expansion activity. The assembled microcontroller module along with the base board is shown in Figure 4.

#### 3.2.1. SD/MMC Card Interface

The SD interface consists of 4 data lines (SDDATA0-SDDATA3) connected to the core module (PA24, PA25, PA22, and PA23) which are in turn connected to the processor through R8, M8, P8, and J9, respectively. Apart from data lines the SD interface has two control signals SDCMD and SDCLK which are connected to K8 and N8. The interface supports DMA transfer. Serial clock line synchronizes shifting and sampling of the information on data lines. The transmission frequency is controlled by making the appropriate bit settings to the SDIPRE register. The frequency can be changed to adjust the baud rate of the connected peripheral. 

#### 3.2.2. Graphic LCD Module

The graphic LCD and touch screen controllers are incorporated in the Samsung microcontroller itself. A 7-inch LC display (Innolux make model AT070TN83) having a resolution of 800X3 (RGB) X480 along with 4 wire resistive touch screen interface is included. The LCD is connected with a forty pin flat ribbon cable. The touch panel is connected using a 4-pin connector. In order to achieve energy savings, a power supply control switch to LCD is incorporated. There is a provision to adjust the brightness also. It displays several messages related to the DAS, GPS/GPRS modem, GPS receiver, and patient details and forms the human machine interface. 

#### 3.2.3. Programming Interfaces

Programming of the embedded system is carried out using JTAG and device USB interfaces. A 10-pin JTAG programming interface is provided on the processor module to program NOR flash using the programming software H flasher. USB device is used for programming the NAND flash that houses the OS and application programs.

#### 3.2.4. Power Supplies

Power supply circuits generate 5 VDC, 3.3 VDC, and 1.8 VDC required for peripherals and other interfaces that include camera, GPS receiver, and USB hub, and so forth. Switched mode regulator 2576 family ICs (LM2576-5 V, LM2576-3.3 V, and LM1117-1.8 for 1.8 V) are employed. RTC battery backup is provided with a 3 V Lithium battery. Battery backup to the unit is provided using a 7.5 V 4000 mAH nickel cadmium battery. A fuel gage circuit incorporating TI make BQ 2019 IC is employed.

#### 3.2.5. Operating System and Embedded Software

Operating systems supported for this board are Linux 2.6 and WinCE 6. The Linux kernel source code used is Linux 2.6.29 and it is configured and cross-compiled using the cross-platform development tool chain arm-Linux-gcc 4.3.2. The drivers for all the peripherals are loaded statically to the kernel. The application code interacts with the hardware device through the device driver associated with the corresponding hardware and accesses the device. 

### 3.3. Peripheral Modules

GPS receiver, camera module, vehicle parameter module, GSM/GPRS/3G module, patient information module, and SOS messaging module are connected to the system using appropriate interfaces as discussed below.

#### 3.3.1. GPS Receiver

The GPS receiver used in the system is a 12-channel GPS receiver module EB818 from Compass Systems. It is built around SiRF-Star III (GSC3f/LP base band processor with integrated flash memory, and RF front end) chipset technology. It is easily integrated in the system being proposed. The module has the advantage of fast acquisition hardware, integrated RF filtering, and a TCXO. GPS receiver is connected through serial interface 1. 

#### 3.3.2. Camera Module

The camera captures the image of the patient and surroundings (in the case of an accident). Logitech/Emprex camera with a resolution of 5 megapixels is connected using USB host 1 interface to the ARM processor. There is a provision to connect a camera with I^2^C interface also. The CMOS image sensor captures the image and forwards it to ARM processor. The CMOS image sensor used is a 1.3 megapixel IC OV9650. It provides the functionality of a single-chip camera and image processor in a small footprint package and outputs full-frame, subsampled, or windowed 8-bit/10-bit images. Timing generator, analog signal processor, A/D converters, digital signal processor, output formatter, and serial camera control bus (SCCB) interface are the main blocks present in the image sensor. Camera is controlled through the SCCB interface and can provide up to 15 frames per second (FPS). SCCB provides a programming interface for all required image processing functions including image quality, formatting, and output data transfer that can be controlled. The image is stored in the micro-SD card. Later the captured image is transferred to the CMS. 

#### 3.3.3. Vehicle Parameter Module

Vehicle parameter acquisition module is built around a low cost PIC family microcontroller 18F452 with a multichannel 10-bit ADC and USB interface for transferring the vehicle data to the microcontroller system. The parameters acquired include fuel level, ignition status, and engine oil level.

#### 3.3.4. GSM/GPRS/3G Module

WAVECOM make GSM/GPRS/3G modem is chosen for transmission of voice, data, and images. Selection of exact model depends upon the service provider. This is connected to the ARM board through RS 232 with a connection speedup to 19,200 bps. In addition to the standard AT commands, the module supports an extended set of AT commands. This facilitates tasks like reading, writing, deleting SMS messages, and sending SMS messages. 

#### 3.3.5. Patient Information Module

KBD2 is a regular QWERTY keyboard connected using USB host 3 to enter administrative and patient information data, mainly alphanumeric. Paramedic staff present in the ambulance can key in any data related to the patient including condition and particulars like his/her name, age, address, and so forth through the keyboard. This message is transmitted to the Central Monitoring Station (CMS) for the medical advice from the doctors. Apart from sending patient information, the staff details like leave, present, or absent can be sent to CMS to notify the authorities.

#### 3.3.6. SOS Messaging Module

KBD1, a 5X5 matrix keyboard, is used to initiate transmission of precanned messages like start of the ambulance from its base station, reaching the site of patient, start of ambulance after picking up the patient, and so forth. Fifteen such messages are provided presently, leaving the other 10 buttons for future expansion. 

#### 3.3.7. Physiological Signal Acquisition Module

**Figure 5:**
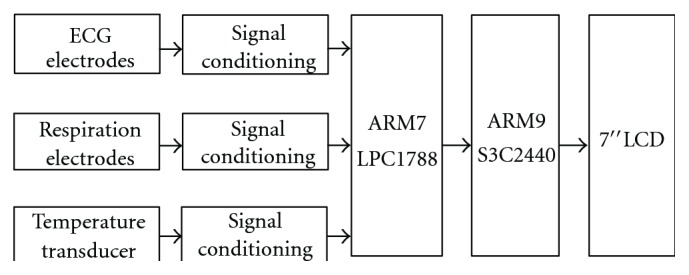
Block diagram of physiological data acquisition system.

The subsystems of this module are shown in Figure 5. 

Physiological data acquisition system acquires the patient's physiological data like ECG, respiration, and temperature through separate modules. The outputs from these modules are connected to an LPC 1788 based data acquisition module through a 200 KSPS, 16-bit resolution, and 8-channel ADC (Linear technologies make LTC1867). They are processed in the system and are transferred to microprocessor based system which in turn transmits to CMS for further processing and action initiation. 

#### 3.3.8. Central Monitoring Station

The CMS consists of several networked computers with voice facility. An accident victim or patient in emergency or an attendant dials a toll free, predesignated number belonging to the central monitoring station being managed by an NGO. The call is answered by the call center executive, who obtains details like the nature of emergency, assesses the help needed, and then transfers the call to a medical expert, emergency response care physician (ERCP) in the call cenetr. Initial advice is given to the patient's attendant. Based on the information related to the location, an ambulance in the vicinity of the site is dispatched. Once the ambulance reaches the site, the emergency medical technician (EMT) provides prehospital care to the victim at the scene. He then connects the physiological data acquisition to the patient, which acquires required data from the victim and transfers the data (including patient's image, previous history, etc.) to the call center ERCP's computer system, where the doctor is available. The data received through internet is extracted to obtain the individual parameters. These extracted parameters include ambulance position, patient's physiological parameters, and patient's image. These are displayed on the graphical user interface of the system. The precise position of the ambulance and its movement is also displayed on the monitor with the help of mapping software on PC at the CMS. Because of this mapping interface the dispatch officer (DO) at the CMS has a chance to know the position of ambulance exactly and guide that ambulance to the accident location on receiving the accident call. Later this software helps the staff at the central monitoring station to guide the ambulance to the appropriate, nearest hospital. EMT is advised by the ERCP, who assesses the patient with the available clinical information made available to him, regarding the care and drugs to be administered to the patient. This is followed by the shifting of the emergency victim to the nearest and appropriate hospital. GPS information is useful in identifying the appropriate hospital with requisite medical care facilities. The ambulance is equipped with a variety of medical equipment, medical consumables, and disposables to ensure the victim is stabilized (prehospital care) by the time the ambulance reaches the hospital for further treatment. Once the patient is handed over to the hospital, the ambulance returns to its predesignated base location and is ready for attending next emergency.

## 4. Testing and Evaluation/Validation

The individual modules of the developed system are tested separately for their independent satisfactory performance. Later they are integrated into a unified system and again tested as a whole.

### 4.1. Power Supply

The power supply is tested for regulation, ripple, load regulation, and line regulation. The required DC voltages are 5.01 V, 3.29 V, and 1.79 V with a ripple content less than 2 mV. 

### 4.2. Signal Conditioners

The ECG signal conditioner is evaluated for gain, frequency response, and CMRR. The values are found to be 1000, 0.03–150 Hz, and 100 db, respectively. The temperature measurement is calibrated using a standard clinical thermometer and water bath for the temperature range 25°C to 50°C. The respiratory module is tested for the impedance variation. As the respiratory activity results in a small change of transthoracic impedance, calibration is done by using a variable resistance. A voltage variation of 0.5 V is obtained for a resistance change of 0.1 Ω for a base resistance of 1500 Ω. 

### 4.3. LPC1788 Board

Clock and reset circuits are tested using an oscilloscope. ADC is tested for a proper conversion by applying variable DC voltage to analog input pins of the board. Specifically developed program reads this data and transfers through USB port to a computer. The data is transferred to a PC through a USB interface. The application running on PC reads this data and displays values on the monitor. Then, a sinusoidal waveform with a varying frequency (0.05 Hz to 200 Hz) is applied and the values are again read and presented on PC screen. This procedure is repeated for all other analog input channels.

### 4.4. Vehicle Module

The parameters monitored in this module are ignition status (digital input), fuel level, and engine oil level (analog inputs). The analog voltages vary from 0 to 3.3 V for minimum to maximum levels. 

### 4.5. GPS Receiver

As the receiver output is available through serial interface, at 4.8 Kbps, the module is tested for accuracy and update frequency by running hyperterminal program on PC.

### 4.6. GSM/GPRS Module

GSM functionality is tested by making a voice call to another cell phone whereas GPRS functionality is tested by connecting to a website on the net.

### 4.7. Camera Module

This module is tested by running the software along with the camera.

### 4.8. Patient Information Module

The patient details as required by the central monitoring station are entered using the QWERTY keyboard and checked for successful transmission.

### 4.9. SD/MMC Card

This card is tested by storing and retrieving typical text files.

### 4.10. Programming Interfaces

**Figure 6:**
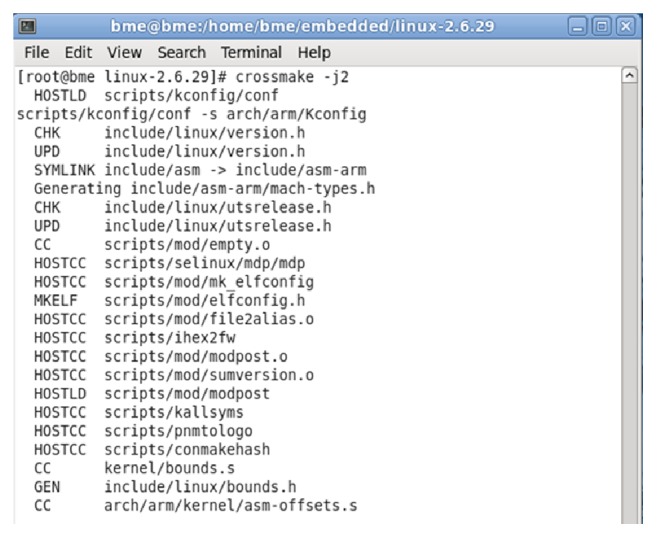
Compilation of the kernel source.

The programming interfaces are tested by connecting serial and USB cables between ARM9 unit and a PC running flash magic and DNW software. Typical snapshots on the PC are indicated in Figure 6.

### 4.11. SOS Messaging Module

 This is tested by pressing the keys on the matrix keyboard for successful transmission to the microcontroller ARM9 unit and subsequent transmission to CMS.

### 4.12. LCD Module

This is tested by transferring LCD test code from PC to ARM9 board and running the program. The module displays a preselected image.

**Figure 7:**
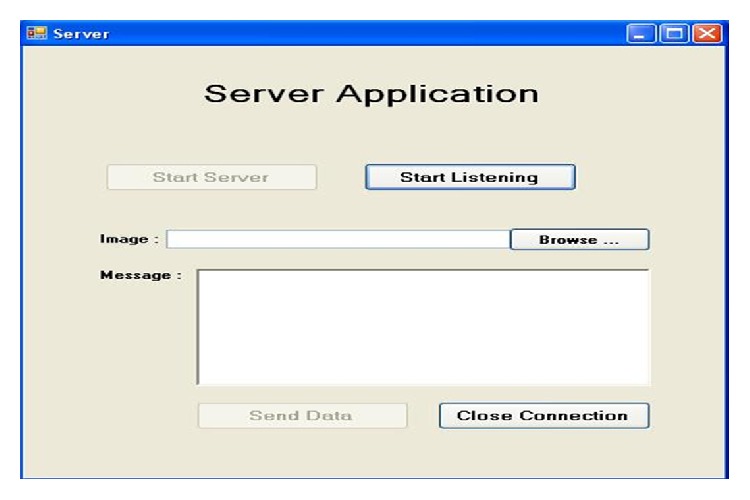
Typical CMS computer screen for testing the connectivity between ambulance unit and CMS.

**Figure 8:**
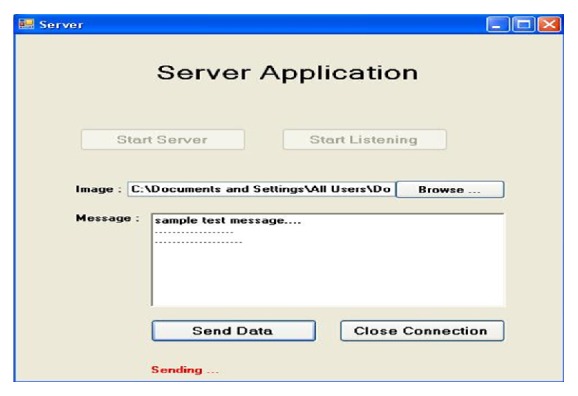
Sample test message transfer from CMS to ambulance unit.

The communication between the ambulance unit and CMS computer is tested using the developed diagnostic software. Typical screenshots are shown in Figures 7 and 8.

## 5. Data Acquisition

An investigation using the developed physiological data acquisition module and GPS receiver has been carried out to explore the possibility of relationship between physiological parameters and geographical location. Two locations with different GPS parameters are chosen for data collection. First location is the Department of Biomedical Engineering, University College of Engineering, Osmania University and the second one is the Rangapur Observatory. The location details are shown in Table 2. 

The signals recorded are ECG, body temperature, and respiration. The recording is repeated three times for the same subject and the average value of these three recordings is considered. Three parameters, namely, heart rate, body temperature, and respiration rate are computed from these recordings. The data are recorded from the subjects of different age groups ranging from nineteen years to fifty-nine years and the mean age is twenty-five years. Students and staff of the Department of Biomedical Engineering, University College of Engineering and Navigational Electronics Research and Training Unit of the Osmania University, volunteered to be subjects. 18 female subjects and 28 male subjects participated as volunteers in this study. 

## 6. Results and Discussion

The base board PCB is designed and fabricated. The circuit is assembled and tested for its power supply functioning as per specifications. The core module is a bought out item. The core module is placed in the connector combination (Figure 8). The unit is tested for power consumption. The serial interface and USB device cables are connected to PC. H-JTAG-Dongle is connected to the J-Tag connector on the core module and the other side to PC's parallel port interface. Using the software H flasher boot loader (Super vivi128.) is loaded into the NOR flash. The boot loader (Super vivi128), kernel (zimage_A70), and file systems (root_qutopia128 M) are subsequently downloaded loaded into the 256 MB NAND flash using DNW software. The application is developed using gnu gcc compiler. Application software, after thorough testing, is also downloaded into the NAND flash.

The use of the processor S3C2440 resulted in compact design of the system. The software modules H-JTAG flasher, DNW, and gnu gcc compilers are employed in the development of software. With user friendly software development tools, the development and change management at site are relatively simpler. The prototype has been installed in an ambulance. The system facilitated acquisition of GPS data (PVT), images, physiological signals, vehicle fuel tank level, keyboard entered administrative data, and SOS messages. The acquired signals are made in to a data packet and sent to a central monitoring station using GPRS. The data are stored locally, when there is connection loss to the internet and transferred as soon as the connection is restored. The application code interacts with camera, data acquisition system, keyboards, GPS receiver, and GSM/GPRS modems through their respective drivers.

**Figure 9:**
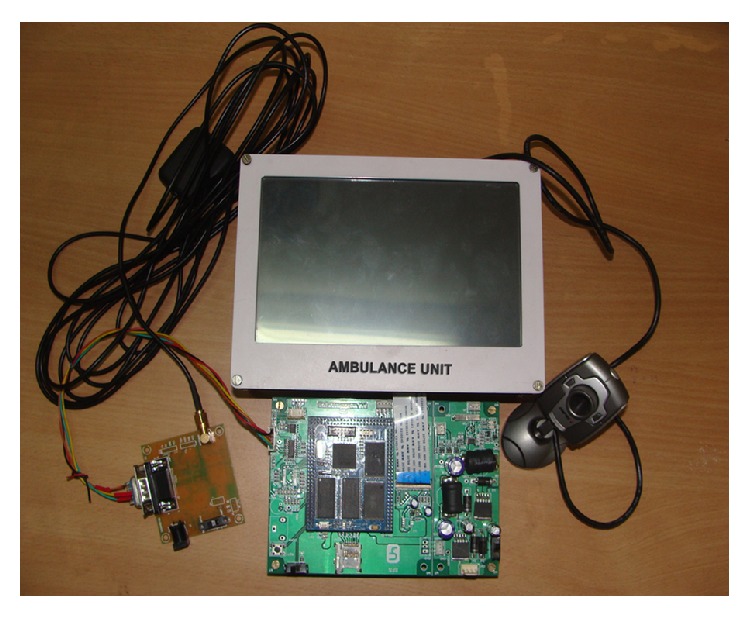
System with all peripheral devices.

**Figure 10:**
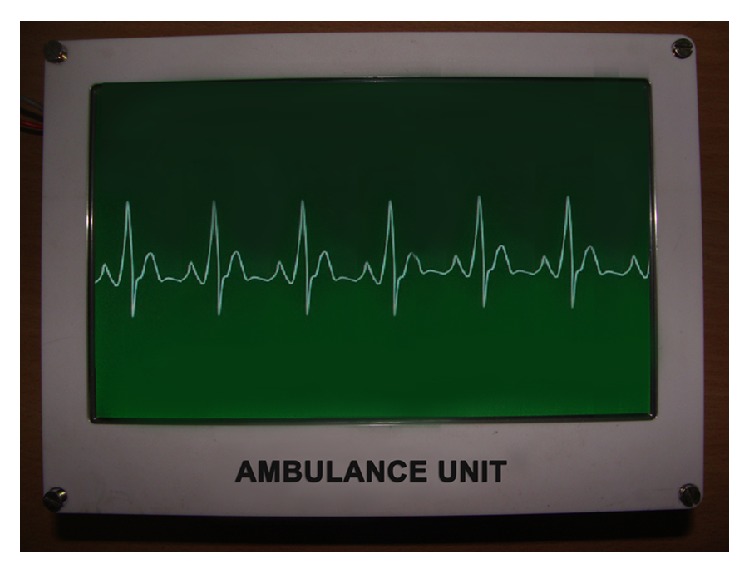
ECG display at the ambulance unit.

The developed ambulance electronics system with all peripherals (Figure 9) is installed inside an ambulance.  Figure 10 indicates the display of ECG in the unit. The image of the surroundings as captured by the camera is also transmitted to the CMS.

### 6.1. Location Based Analysis of Physiological Parameters

**Figure 11:**
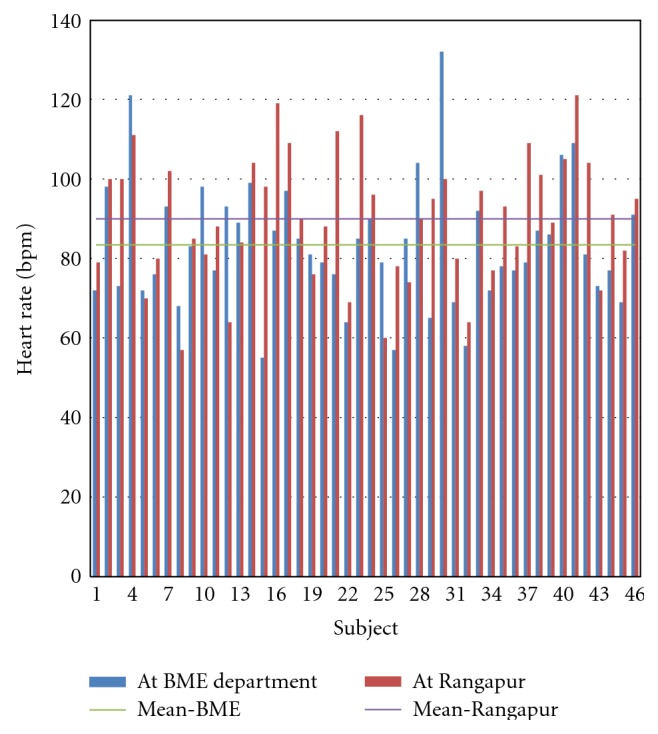
Heart rate of all subjects at the two locations.

 It is clearly noted that heart rate increased at Rangapur Observatory compared to that at the Department of BME. The mean heart rate increased from 83 at the Department of BME to 90 at Rangapur Observatory. Thus the percentage increase in heart rate between these two locations is 8. Out of the 46 subjects, the heart rate increased for 33 subjects and it decreased for 13 subjects (Figure 11). The maximum increase is 43 beats per minute and the minimum increase is 2 beats per minute; while the maximum decrease is 32 beats per minute, the minimum decrease is 1 beat per minute. 

**Figure 12:**
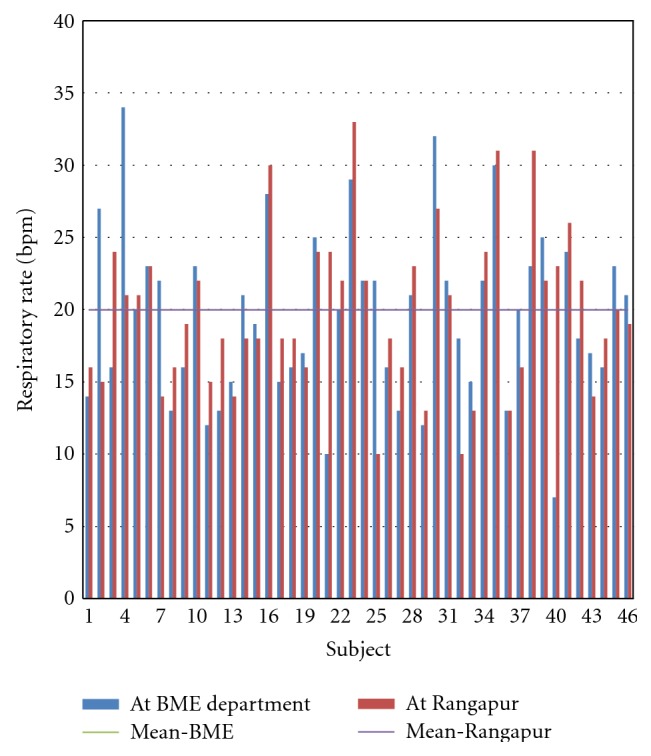
Respiratory rate of all subjects at the two locations.

The respiratory rate of 23 subjects increased and that of 23 subjects decreased. The increase in respiratory rate ranged from 1–14 breaths per minute while the decrease ranged from 1–13 breaths per minute. As seen from Figure 12, there is no change in the mean value of respiratory rate between the two locations.

A slight variation in the parameters is observed between female and male subjects. Though the mean values of heart rate, respiration rate of female subjects are slightly higher than those of male subjects at both locations, the difference between male and female subjects is different at the two locations as shown in Table 3. There is no significant change in the body temperature between the two locations. 

## 7. Conclusion

The ambulance electronics system consisting of ARM 9 microcontroller has been designed, fabricated, and tested. The prototype has proved that it is possible to integrate and deploy an integrated medical data acquisition system, administrative data collection system, and communication system that is cost effective as well as life saving. An engineered model based on this prototype can be built and installed in the ambulances engaged for patient transportation. Engineered models, when installed in the ambulances, will enhance the capability of emergency patient transportation organizations in terms of reduction in time to reach the nearest hospital and extending timely prehospital medical care. The features of the system can be enhanced to meet the requirements from time to time based on the experience gained while using the system.

There has been a significant change in the heart rate of the subjects between the two locations. The changes in physiological parameters might be attributed to the difference in altitude (about 150 meters) between the two locations. As the data has been recorded from a small and healthy group of subjects, further work on a much larger database is required to establish the results obtained in this study regarding the effect of location on physiological parameters. Hence to derive more useful conclusions, larger group of subjects, both normal and abnormal, with different age groups have to be studied. This work can be extended by taking the recordings at different geographical locations. Diagnostically significant information could be extracted and the effect of location (as obtained by GPS receiver) on physiological parameters could be identified more successfully. Critically ill patients can be warned about moving to high altitude locations.

The system developed here is a product of extensive need-based research projected by government and nongovernment organizations engaged in emergency patient transportation. Different manufacturers provide data in different formats. One cannot employ systems from different manufacturers in the same project, as interoperability becomes difficult. They do not provide the data that can be easily read by scientific software for further processing. The system, as it provides data in text format, alleviates all such problems. Such a system will also avoid dependence on proprietary software to store and process the data. 

## Figures and Tables

**Table 1:** Typical specifications of the proposed system.

Operating voltage	12 V DC
Operating current	400 mA
GPS receiver update	Every one second
GPRS modem frequency	900/1800 MHz
LCD display	800 × 600 pixel 7′′
Camera	1 M pixel
Serial interfaces	3
USB interfaces	3
Programming interfaces	1 USB + 1 serial
Parameters monitored	ECG, respiration, temp.

**Table 2:** GPS parameters of the two locations.

Location	Latitude	Longitude	Altitude
Department of BME	17° 24′ 32.858	78° 31′ 09.903	451.77 m
Rangapur observatory	17° 05′ 45.024	78° 43′ 04.695	605.74 m

**Table 3:** Gender-wise physiological parameters at the two locations.

Gender	Department of BME			Rangapur		
	Heart rate (beats/min)	Temp. (°C)	Resp. (breaths/min)	Heart rate (beats/min)	Temp. (°C)	Resp. (breaths/min)
Female	85	37	20	95	37	20
Male	82	37	19	87	38	20

## References

[2] Yen Y. S., Chiang W. C., Hsiao S. F., Shu Y. P. Using WiMAX network in a telemonitoring system.

[3] Liszka K. J., Mackin M. A., Lichter M. J., York D. W., Pillai D., Rosenbaum D. S. (2004). Keeping a beat on the heart. *IEEE Pervasive Computing*.

[4] Plesnik E., Malgina O., Tasič J. F., Zajc M. ECG signal acquisition and analysis for telemonitoring.

[5] Khan A., Mishra R. (2012). GPS–GSM based tracking system. *International Journal of Engineering Trends and Technology*.

[6] Zhang J., Lu Z. The mobile ECG telemonitoring system based on GPRS and GPS.

[7] Zhang J., Lu Z. The mobile ECG telemonitoring system based on GPRS and GPS.

[8] Fang Z.-X, Lai D.-K (2007). Uninterrupted ECG mobile monitoring. *International Journal of Bioelectromagnetism*.

[9] Alesanco Á., García J. (2010). Clinical assessment of wireless ECG transmission in real-time cardiac telemonitoring. *IEEE Transactions on Information Technology in Biomedicine*.

[10] Krejcar O., Slanina Z., Stambachr J., Silber P., Frischer R. Noninvasive continuous blood pressure measurement and GPS position monitoring of patients.

[11] Kiss N., Patai G., Hanák P. Vital fitness and health telemonitoring of elderly people.

[12] Yedukondalu K., Sarma A. D., SatyaSrinivas V. (2011). Estimation and Mitigation of GPS Multipath Interference using Adaptive filtering. *Journal of Progress in Electromagnetics Research M (PIER M), U.S.A.*.

[13] Venkata Ratnam D., Sarma A. D., Satya Srinivas V., Sreelatha P. (2011). Performance evaluation of selected ionospheric delay models during geomagnetic storm conditions in low-latitude region. *Radio Science*.

